# Viscoelastic Separation and Concentration of Fungi from Blood for Highly Sensitive Molecular Diagnostics

**DOI:** 10.1038/s41598-019-39175-5

**Published:** 2019-02-28

**Authors:** Jeonghun Nam, Woong Sik Jang, Da Hye Hong, Chae Seung Lim

**Affiliations:** 1 Department of Laboratory Medicine, College of Medicine, Korea University Guro Hospital, Korea University, Seoul, Korea; 2 Department of Emergency Medicine, College of Medicine, Korea University Guro Hospital, Korea University, Seoul, Korea

## Abstract

Isolation and concentration of fungi in the blood improves sensitivity of the polymerase chain reaction (PCR) method to detect fungi in blood. This study demonstrates a sheathless, continuous separation and concentration method of candida cells using a viscoelastic fluid that enables rapid detection of rare candida cells by PCR analysis. To validate device performance using a viscoelastic fluid, flow characteristics of 2 μm particles were estimated at different flow rates. Additionally, a mixture of 2 μm and 13 μm particles was successfully separated based on size difference at 100 μl/min. Candida cells were successfully separated from the white blood cells (WBCs) with a separation efficiency of 99.1% and concentrated approximately 9.9-fold at the center outlet compared to the initial concentration (~2.5 × 10^7^ cells/ml). Sequential 1st and 2nd concentration processes were used to increase the final number of candida cells to ~2.3 × 10^9^ cells/ml, which was concentrated ~92-fold. Finally, despite the undetectable initial concentration of 10^1^ CFU/ml, removal of WBCs and the additional buffer solution enabled the quantitative reverse transcription (RT)-PCR detection of candida cells after the 1st concentration (Ct = 31.43) and the 2nd concentration process (Ct = 29.30).

## Introduction

*Candida albicans* (*C. albicans*) is an opportunistic pathogen and is a leading cause of bloodstream infections in hospitals. It results in a 40–54% mortality rate in patients with Candidemia^1,2^. Early and accurate diagnosis of invasive fungal infections in the blood stream is crucial to managing Candidemia by rapid antifungal therapy^3^. Using a blood culture is still considered an optimal standard for the diagnosis of invasive candidiasis^4^. However, it is difficult to cultivate an extremely low concentration fungi in blood (<10 CFU/ml) and generally 2–4 days are required for blood culture prior to obtaining a positive diagnosis^4^, which can be potentially fatal. In order to address these limitations, various studies were conducted to develop non-culture-based diagnosis methods such as mannan detection, (1–3)-β-d-glucan (BDG) detection, immunomagnetic detection, and polymerase chain reaction (PCR). Immunity-based diagnostic methods using BDG exhibited low sensitivity (40–100%) and specificity (45–99%)^5^ and a mannan assay was unable to detect *Candida parapsilosis* and *Candida guilliermondii*, despite sensitivity and specificity of approximately 80% and 85%, respectively^6^. Despite the frequent use of immunomagnetic detection, this method has been limited by a rather low recovery rate and requirement of a labeling process^7,8^. Meanwhile, real-time PCR is considered to be the most developed diagnostic method, shown in a recent meta-analysis to have high sensitivity (95%) and specificity (92%)^9^. However, the detection of low concentration fungal pathogens in blood is still limited to PCR due to the analytical sensitivity of the assay (3–100 CFU/ml)^10^. Recent studies indicate that *in vitro* detection limits were at least 4 CFU/ml in blood^11^ and 3 CFU/ml in blood^12^.

It is expected that the detection of extremely low concentration fungi in blood could be significantly improved by removing nonspecific binding with fungal primers/probes of DNA contaminants of other nucleated blood cells in the sample^13–15^. For example, white blood cells (WBCs) can affect the detection sensitivity of PCR analysis, which has a normal count between 4 × 10^3^/μl and 1.1 × 10^4^/μl. On the other hand, platelets of similar size (approximately 2–3 μm) with candida cells are not considered in PCR analysis despite a large population between 2 × 10^5^/μl and 5 × 10^5^/μl, due to the lack of nuclear DNA, yet they contain a significant amount of microRNA^16^. More recently, a chemical lysis method has been introduced to lyse every blood cell and remove PCR inhibitors efficiently without degrading fungal cell walls^12^. However, an extremely low fungal concentration still limits high-sensitivity and high-specificity detection. Therefore, methods for separation and concentration of fungal cells needs to be developed.

Significant advancements in microfluidic technology over the past two decades led to an increased use of microfluidic-based separation and concentration of particles/cells from a heterogeneous mixture sample in biological, chemical, environmental, and clinical applications^17–20^. The strategy of using microfluidic devices for sample preprocessing has been widely applied before post-analysis such as in flow cytometry, PCR, surface plasmon resonance (SPR), and spectrophotometry^14,21–25^. Microfluidic separation and concentration techniques are divided into two categories based on the use of the following external force fields: active and passive techniques. Active techniques employ various force fields, including electric^26,27^, magnetic^28,29^, optical^30,31^, and acoustic fields^32–35^ to manipulate particles/cells, while passive methods rely only on channel geometry and/or hydrodynamic effects of the flow^36,37^. Passive methods eliminate the need for any external force fields, although several of them require an elaborate channel design and high flow rate conditions for effective particle/cell manipulation.

Viscoelastic non-Newtonian microfluidics gained considerable attention due to intrinsic nonlinear elastic forces in pressure-driven flows of polymer solutions^14,38–46^. Compared to previous passive methods, such as inertial microfluidics performed in Newtonian fluid, viscoelastic particle/cell manipulation can be achieved over a wide range of flow rates. In addition, the separation target size can be adjusted by simply changing the viscoelastic properties of the suspending medium and the flow rate. The non-uniform distribution of the first normal stress difference (*N*_1_) in a non-Newtonian fluid can laterally drive suspended particles/cells into a simple straight microchannel. This was applied to three-dimensional particle focusing^38^ and particle separation based on size differences^40^. Before the separation process, the lateral position of all particles/cells was initialized by using sheath flow to enhance the separation efficiency. However, the use of sheath flow limits multi-processing of parallel channels, which decreases the device throughput and dilutes the concentration of particles/cells. Recently, rather than using sheath flow, specific geometries were used to focus particles/cells. Asymmetric grooved geometries should be used with careful consideration to generate Dean flows for three-dimensional particle/cell focusing^47,48^.

Meanwhile, in our previous work, the circular shaped channel or high aspect ratio (*AR* = height/width) microchannel were used to align particles/cells along the inner walls at the bifurcation channel without the aid of sheath flow before the separation process^14,49,50^. Using these devices, cells of different sizes could be separated with high efficiency and high purity, however, the channel dimension should be carefully determined considering the lateral displacement of target cells in order to achieve high separation efficiency. Additionally, in these devices, the flow at the bifurcation channel can affect the shear-sensitive cells. More recently, label-free and sheathless particle/cell separation in a straight microchannel with a low AR was demonstrated using a poly (ethylene oxide) (PEO) solution^51–54^. Particles laterally migrated toward the size- and shape-dependent equilibrium positions. However, the device throughput for particle separation was limited to ≤50 μl/min^51^ and ≤5 μl/min^53^ due to the flow rate-dependent elasto-inertial flow characteristics. In addition, the flow resistance in the microchannel was proportional to the dynamic viscosity of the fluid, PEO solution^55^, which affected the device throughput. The devices described above have focused only on particle/cell separation to enhance the purity, however, the concentration of target cells was still not high enough for sensitive detection. To address the limitations of previous research, a microfluidic device that enables simultaneous cell separation and concentration with high throughput is required.

In this study, we proposed a sheathless, label-free candida cell separation and simultaneous concentration by depleting WBCs from a lysed whole blood sample in a low-AR microchannel with high efficiency and high throughput. To achieve a high throughput, a viscoelastic polymer solution with low viscosity was used. To the best of the authors’ knowledge, previous studies have not used microfluidic devices for continuous separation from nucleated blood cells and simultaneous concentration of candida cells for ultrasensitive PCR detection. We examined the flow characteristics of particles with different sizes and equilibrium position-based particle separation was demonstrated, negating the need for initialization of all the particles. Finally, the same flow characteristics in our device were adopted to separate and concentrate candida cells for clinical diagnosis. The device performance was validated by post-analyses using flow cytometry and PCR analysis. Our device can be utilized for pre-processing before not only PCR analysis but also various post-analysis techniques, including surface plasmon resonance (SPR) and spectrophotometry.

## Results

### Working principle

The characteristics of flow and particle migration in the viscoelastic flow are defined by non-dimensional numbers. The Reynolds number (*Re*) describes the ratio of the inertial force to the viscous force while the Weissenberg number (*Wi*) describes the ratio of the elastic force to the viscous force as follows:

1 $$Re=\frac{\rho {V}_{m}{D}_{h}}{{\eta }_{c}}$$

2 $$Wi={\rm{\lambda }}{\dot{\gamma }}_{c}$$

here, $\rho$, *V*_*m*_, *D*_*h*_, *η*_*c*_, *λ*, and ${\dot{\gamma }}_{c}$ denote the solution density, mean flow velocity, hydraulic diameter of the particle, characteristic viscosity of the solution, fluid relaxation time, and characteristic shear rate, respectively. The particles suspended in the viscoelastic fluid experience the simultaneous effect of elastic and inertial lift forces. Therefore, the relative effect of fluid elasticity to inertia is estimated by using the elasticity number (*El* = *Wi/Re*).

The elastic lift force (*F*_*e*_) is induced by non-uniform differences in the first normal stress (*N*_1_) which create additional tension along the flow streamlines as follows^38^:

3 $${F}_{e} \sim {a}^{3}\frac{\partial {N}_{1}}{\partial x} \sim \lambda {(a/W)}^{3}{Q}^{3}$$

Here, *a*, *x*, *W*, and *Q* denote the particle diameter, lateral distance, width of the microchannel, and volumetric flow rate, respectively. The elastic force drives the suspended particles to low shear rate regions at the centerline and the corners of the microchannel. Conversely, the inertial lift force also involves the lateral migration of particles in a viscoelastic fluid. The inertial lift force has two counteracting components, the shear-gradient lift force toward the channel walls (*F*_*i,s*_), and the wall repulsion force toward the channel center (*F*_*i,w*_). The shear-gradient lift force diminishes to zero near the corners of the microchannel and the wall repulsion force becomes dominant. The expression is as follows:

4 $${F}_{i}={F}_{i,s}+{F}_{i,w} \sim \rho {(a/W)}^{4}{Q}^{2}$$

As shown in Eq. (3) and (4), the elastic lift force and the inertial lift force are substantially affected by the particle diameter.

**Figure 1:**
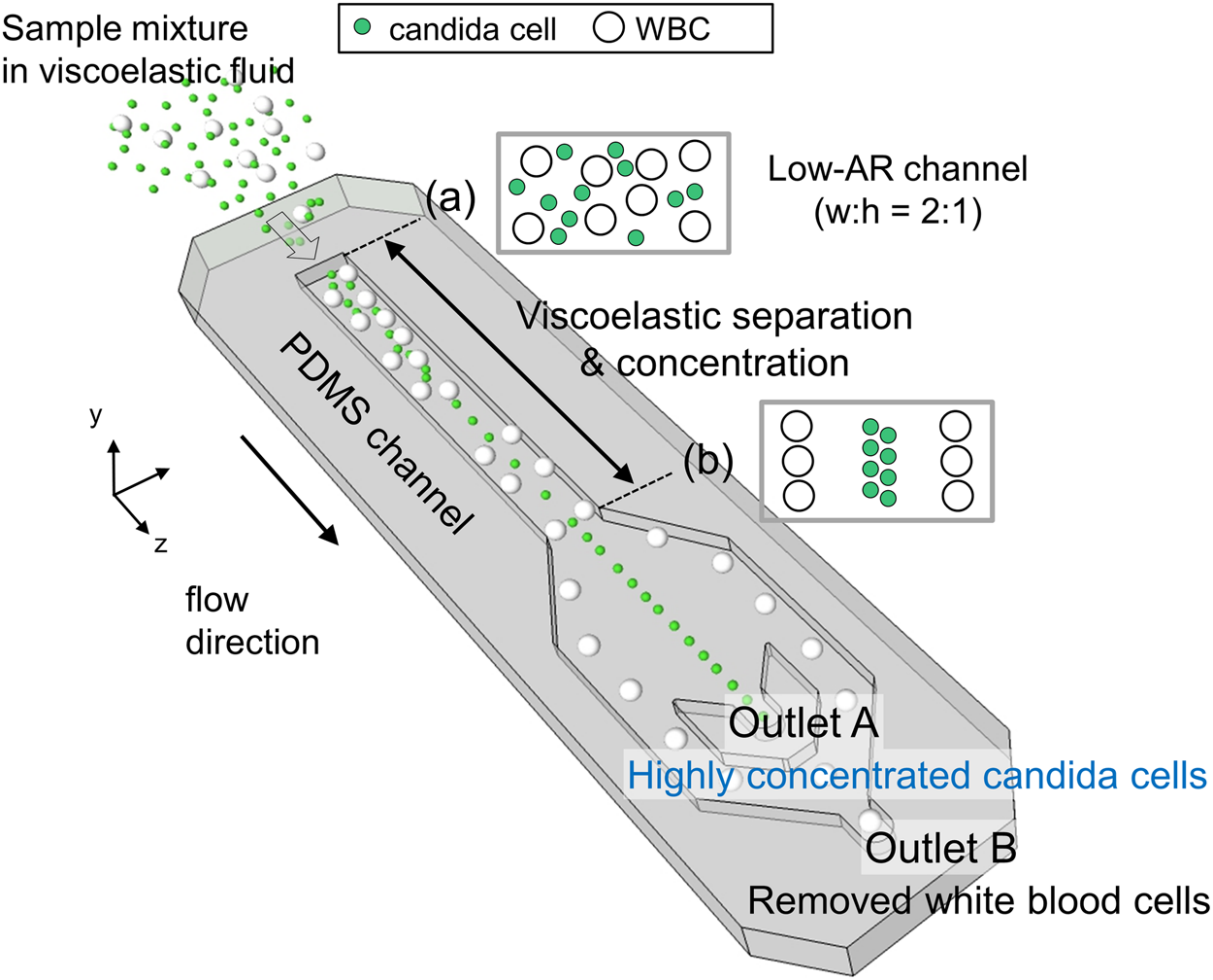
Schematic of continuous viscoelastic separation and concentration of candida cells. (**a**) Sample mixture containing WBCs and candida cells in a viscoelastic fluid were randomly introduced to the inlet. Candida cells were separated and concentrated at outlet A due to size-dependent viscoelastic separation while WBCs were removed at outlet B in a low aspect ratio microchannel.

A schematic of the device for sheathless viscoelastic particle focusing and separation is shown in Fig. 1. A sample mixture containing small candida cells and relatively larger WBCs was injected at the inlet and the cells were randomly distributed (Fig. 1(a)). With respect to small cells (candida cells) relative to the channel size in the low-AR channel, inertial lift force drove cells away from the channel walls and the center while the elastic lift force drove the particles to the centerline of the channel. However, with respect to the cells with a relatively high blockage ratio (*β* = *a/H*, *H* denotes the channel height), elastic normal stresses drove the particles toward the channel walls, which was different from the cells with a low blockage ratio. Therefore, candida cells were tightly focused at the center of the microchannel while WBCs migrated toward the two equilibrium positions between the channel center and the side walls (Fig. 1(b)). Finally, highly concentrated candida cells were collected at the center outlet (Outlet A) while WBCs were eliminated from the initial sample mixture toward the rear outlet (Outlet B).

### Effect of viscoelasticity on flow characteristics

**Figure 2:**
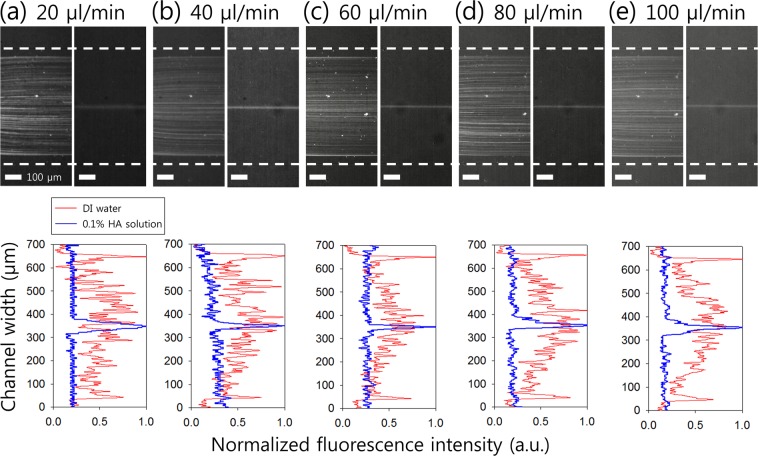
Effect of viscoelasticity on the flow rate-dependent flow characteristics of 2 μm fluorescent particles suspended in DI water (left) and 0.1 (w/v)% HA solution (right). (Top) Stacked microscopic images and (Bottom) normalized fluorescent intensity in an expansion region at flow rates of (**a**) 20 μl/min, (**b**) 40 μl/min, (**c**) 60 μl/min, (**d**) 80 μl/min, and (**e**) 100 μl/min. Dotted white lines indicate the channel sidewalls.

In order to examine the effect of viscoelasticity on flow characteristics of 2 μm fluorescent particles (*β* = 0.08), flow rate-dependent distributions of particles suspended in deionized (DI) water and 0.1% HA solution were observed over a wide range of flow rates from 20 μl/min (*Re* = 9.87, *Wi* = 2.77, *El* = 0.28) to 100 μl/min (*Re* = 49.38, *Wi* = 13.86, *El* = 0.28). Figure 2 shows the normalized fluorescence intensity in the expansion region during the flow of 2 μm particles. In DI water, 2 μm particles were randomly distributed in the microchannel over a wide range of flow rates from 20 μl/min to 100 μl/min. However, 2 μm particles suspended in 0.1% HA solution were focused into a single band along the channel center due to the elastic lift force toward the center.

### Effect of the particle size on flow characteristics

**Figure 3:**
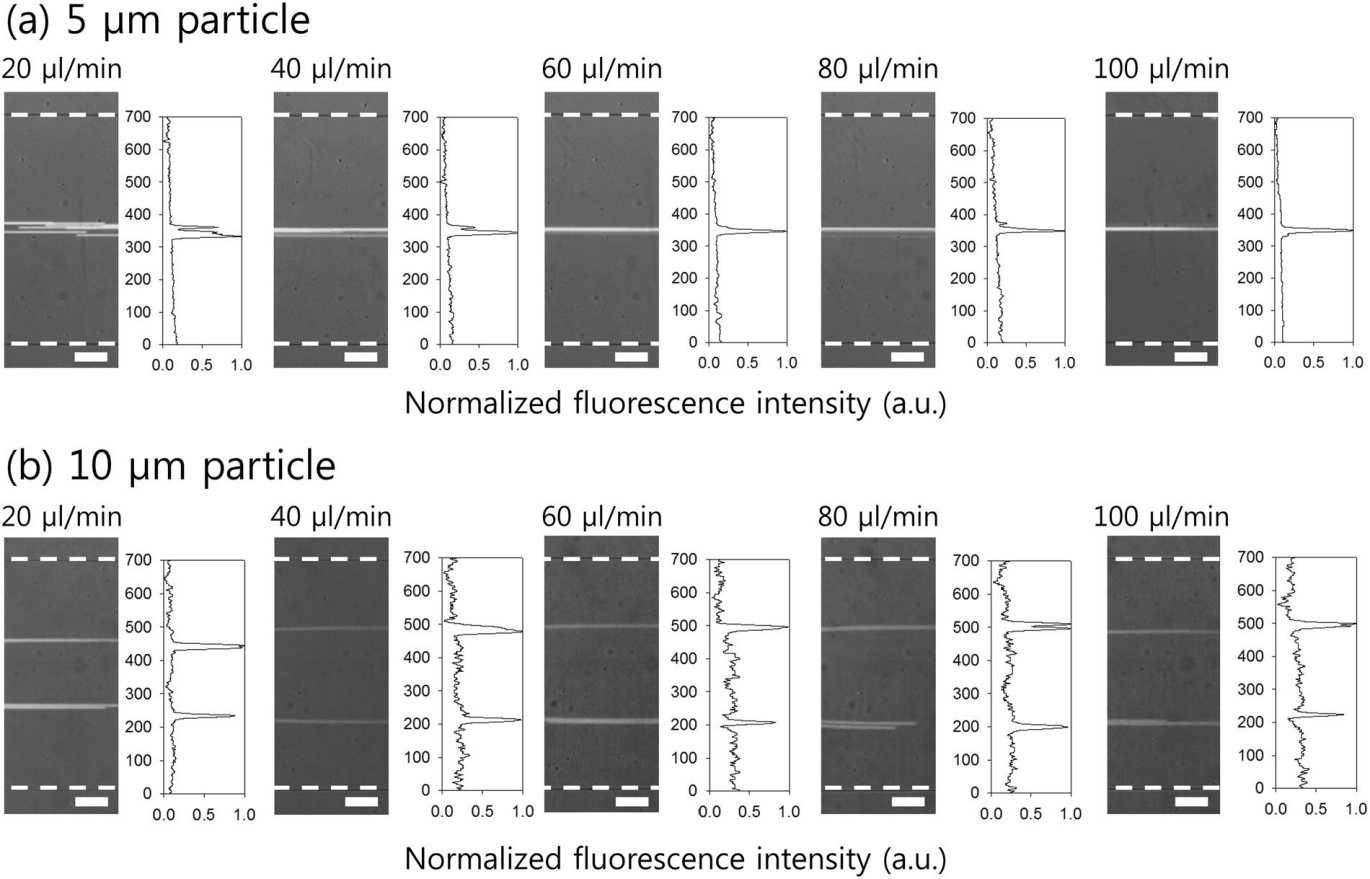
Effect of particle diameter on the flow rate-dependent flow characteristics of fluorescent polystyrene particles with (**a**) 5 μm diameter and (**b**) 10 μm diameter. Stacked microscopic images (left) and normalized fluorescent intensity in an expansion region (right) at different flow rates of 20 μl/min, 40 μl/min, 60 μl/min, 80 μl/min, and 100 μl/min. The dotted white lines indicate the channel sidewalls.

Flow rate-dependent particle distributions in the expansion region were monitored with varying flow rates between 20 μl/min and 100 μl/min by using particles with diameters of 5 μm (*β* = 0.2) and 10 μm (*β* = 0.4) in 0.1% HA solution. Figure 3 shows that 5 μm particles (*β* = 0.2) were focused along the centerline while 10 μm particles (*β* = 0.4) were focused into two fluorescent streams at both sides of the centerline in the entire range of flow rates (20–100 μl/min). An increase in the flow rate increased the *Wi*, i.e., elastic force increased, and suspended particles were more tightly focused at the equilibrium positions. These results indicated significant consistency with previous studies in which off-center focusing into two streams was reported in numerical studies (*β* ≥ 0.25) and microfluidic experiments involving the use of viscoelastic fluid (*β* = 0.3 with *El* = 0.028 and 0.11). Flow rate-dependent distributions of smaller particles (1 μm diameter, *β* = 0.04) were also examined (Fig. S1).

The equilibrium position of particles was determined by the simultaneous effect of flow inertia and flow elasticity, shear thinning effect during the flow, and blockage ratio of each particle in the microchannel. The off-center focusing of large particles (*β* = 0.4) was attributed to the elastic lift force that acted on the side walls (*F*_*el,w*_). The elastic lift toward the wall was due to fluid elasticity and shear thinning effects, and was strongly dependent on particle size. Therefore, the equilibrium positions of large particles were pushed toward side walls and off-centered. Our device can be applied to sheathless and label-free particle/cell separation based on the size-dependent equilibrium positions during the flow.

### Separation of binary mixtures of particles

**Figure 4:**
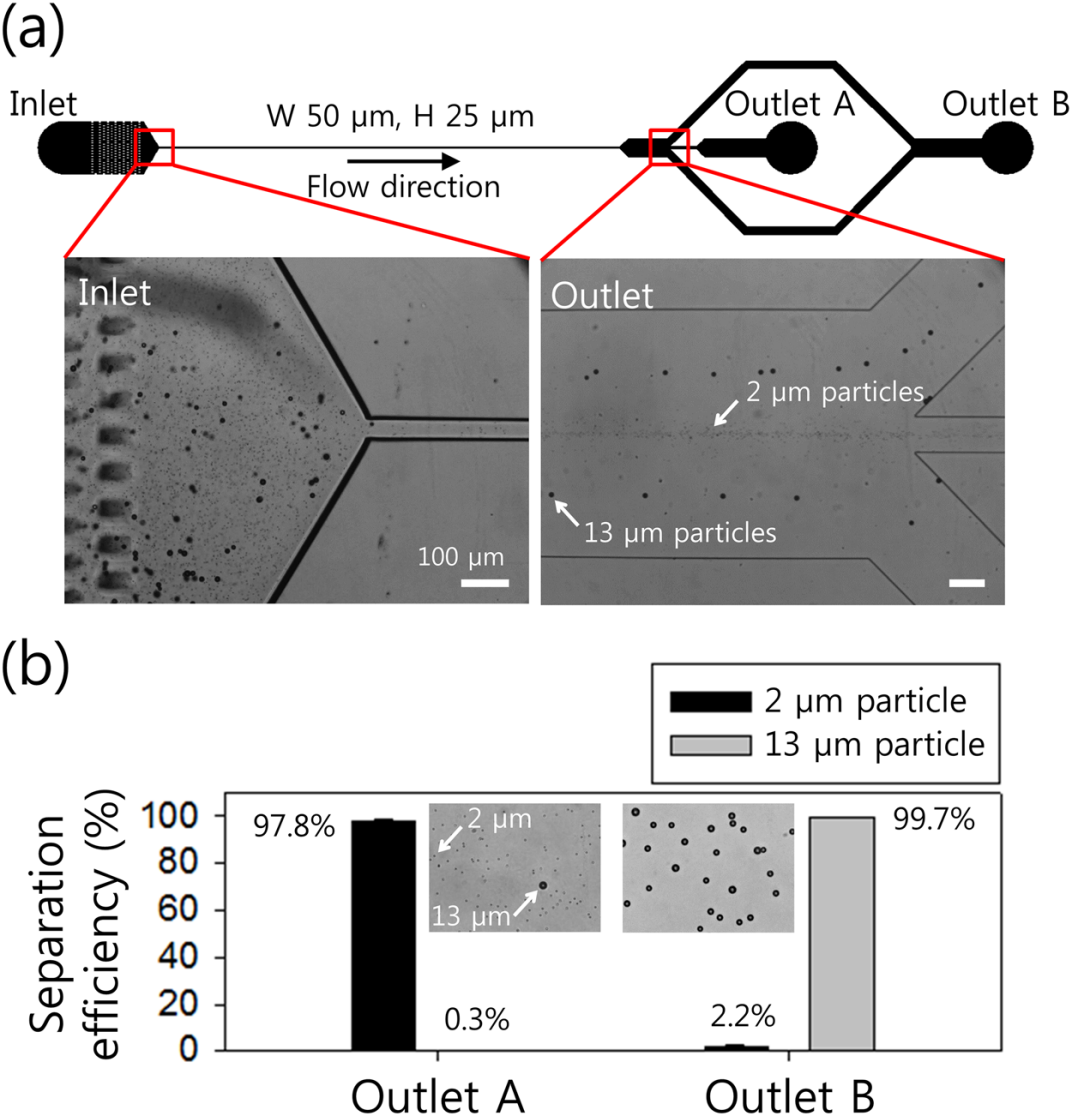
Separation of the binary mixture of 2 μm and 13 μm particles at a flow rate of 100 μl/min. (**a**) A stacked microscopic image indicates that randomly injected particles at the inlet were separated into two different streams along the center (2 μm particles) and off-center (13 μm particles). (**b**) Separation efficiency at each outlet by using the collected sample after the separation process. Collected particles from outlets A and B are shown. The standard deviation depicts the measured values from five different experiments (*n* = 5).

Figure 4 shows the viscoelastic separation of binary mixtures of 2 μm (*β* = 0.08) and 13 μm particles (*β* = 0.52) in 0.1% HA solution at a fixed flow rate of 100 μl/min (*Re* = 49.38, *Wi* = 13.86). A stacked microscopic image at the channel inlet (Fig. 4(a), left) shows a random distribution of both 2 μm and 13 μm particles. At the outlet expansion, three distinct streams were formed with the outer two streams of 13 μm particles and a single stream of 2 μm particles at the channel center. The outer two streams of 13 μm particles approximately corresponded to 1/4 of the channel width from the channel sidewalls and flowed to the side outlet channels. Furthermore, 2 μm particles were separated from larger particles and collected at outlet A. The microscopic images in Fig. 4(b) show the sample collected from each outlet. In outlet A, approximately 97.8% of 2 μm particles were collected with high purity (approximately 99%) while approximately 99.7% of 13 μm particles were collected from outlet B.

### Effect of viscosity of a lysed blood sample on separation performance

The separation of candida cells from WBCs was performed to examine the potential of our device for clinical application. The viscosity of a lysed blood sample with a hematocrit of 50 was known to be higher than that of blood plasma (~2.4 cP)^56^, which could affect the size-dependent lateral migration to the equilibrium position by the flow inertia and flow elasticity. The effect of the increased viscosity of the suspension was examined using viscous aqueous glycerol solution containing 2 and 13 μm particles (Fig. S2). In glycerol solution with the viscosity between 1 and 2.5 cP, the streamline of 2 and 13 μm particles was briefly almost similar to those in Figs 2 and 4. Separation of 2 and 13 μm particles was successfully achieved, even from a high viscosity suspension at ~2.5 cP. Therefore, we found that the viscosity of the lysed blood sample would not affect the device performance of separation and concentration of candida cells from WBCs in a lysed blood sample. However, for further use of lysed blood samples, possible biological effects of plasma proteins on the device performance should be considered.

### Separation of candida cells from WBCs

**Figure 5:**
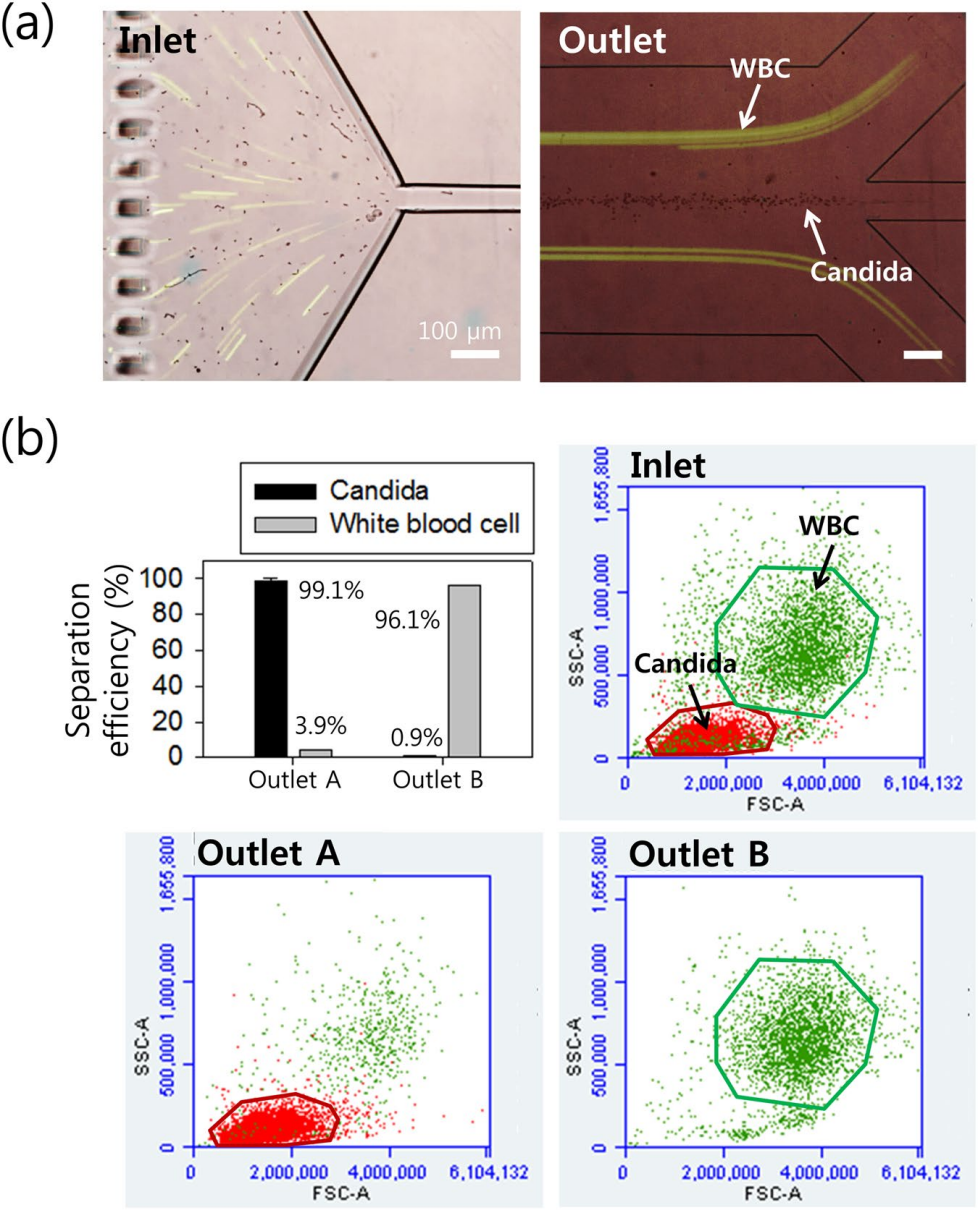
(**a**) Cell separation for the WBCs and candida sample at a flow rate of 100 μl/min. Bright-field microscopic images (candida) and fluorescent images (WBCs) were stacked to show the separated streamlines of each cell at the outlet region. (**b**) Flow cytometric scattergrams and separation efficiency after the separation process. Prior to the separation, the binary mixture contained the WBCs and candida. Most unwanted WBCs were successfully removed from the injected sample at outlet B while several candida were collected at outlet A. The standard deviation of the separation efficiency depicts the measured values from five different experiments (*n* = 5).

Figure 5(a) shows the microscopic images at the inlet and outlet of the microchannel during the separation process superimposed from bright-field images for candida and fluorescent images for WBCs at the fixed flow rate of 100 μl/min. At the inlet, a binary mixture containing both cells was randomly distributed in the microchannel. At the outlet, candida and WBCs exhibited separate streamlines flowing to different outlets. Candida was tightly focused at a single equilibrium position in the center of the microchannel due to relatively small sizes (approximate diameter of 3 μm, *β* = 0.12), while the equilibrium positions of WBCs were off-center shifted toward the side walls. According to the flow characteristics of particles in Figs 2 and 3, particles larger than 10 μm (*β* ≥ 0.4) were clearly removed at outlet B. The diameter of WBCs was 9–15 μm (0.36 ≤ *β* ≤ 0.6). Fluorescent streamlines of WBCs were approximately 1/3 of the channel width from the channel sidewalls, and this was similar to the equilibrium positions of 13 μm particles.

Flow cytometric analysis was conducted to evaluate the separation performance of the device. Additionally, 10,000 events were counted with preset gates determined by each sample and cell distribution was achieved, as shown in Fig. 5(b). Prior to the separation, the binary mixture sample contained WBCs and candida. After the separation, candida was successfully extracted at outlet A and most WBCs were removed at outlet B. Separation efficiency is the ratio of the number of target particles at the target outlet to the total number of particles found at both outlets. In outlet A, 99.1% of the desired candida was collected from the total number flowing through both outlets while 96.1% of unwanted WBCs were removed at outlet B. The purity of candida collected at outlet A was approximately 97%, and this is defined as the ratio of the number of target particles to the total number of particles collected at outlet A. The results indicate that WBCs at outlet B exhibited slightly lower separation efficiency compared to that of 13 μm particles. This might be due to the heterogeneous size distribution and deformability of WBCs. However, a slight reduction in separation performance did not significantly affect the separation of candida cells, and this was verified by the PCR analysis.

### Enhancement of concentration factor

**Figure 6:**
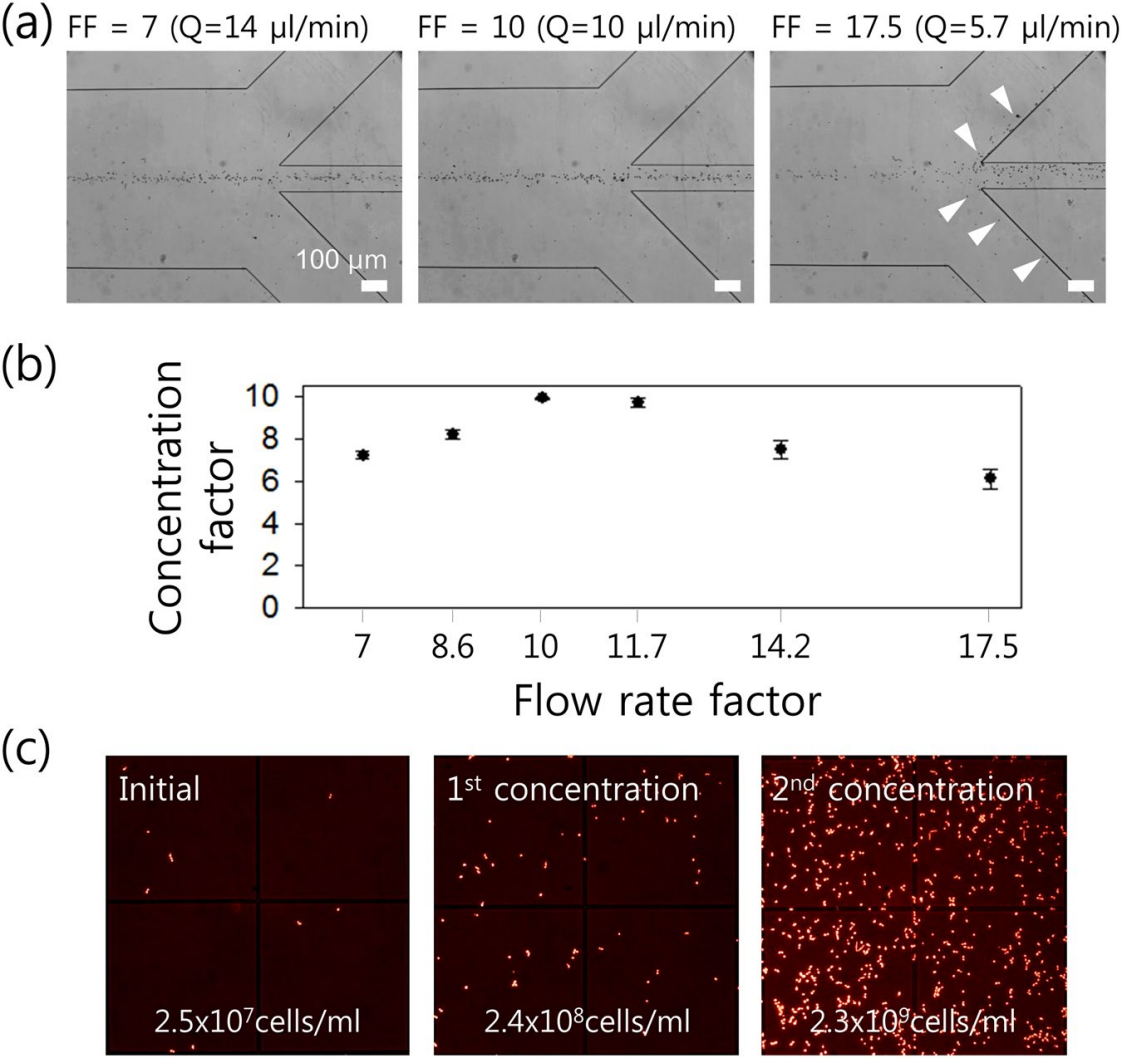
(**a**) Concentration of candida based on different flow rate factors determined by the suction flow rate from outlet A. (**b**) Concentration factor at various flow rates. (**c**) Fluorescent-dyed candida in the inlet (2.5 × 10^7^ cells/ml) and collected from outlet A after the 1st concentration process (2.4 × 10^8^ cells/ml) and the 2nd concentration process (2.3 × 10^9^ cells/ml).

During the separation process, candida could also be concentrated by removing additional suspending medium at outlet B. To enhance the concentration of candida cells suspended in a lysed blood sample, the suction flow rate at outlet A could be controlled to remove additional buffer solution at outlet B. The flow rate factor (FF) is the ratio of the inlet flow rate to the outlet flow rate at outlet A of the trifurcation outlet region^57^. With respect to the separation process, the inlet flow rate was fixed at 100 μl/min while the outlet flow rate at outlet A was determined by the ratio of the channel width at the trifurcation region. In this study, the widths of the outlet channels were designed as 300 μm, 100 μm, and 300 μm, respectively, such that the initial flow rate factor was determined to be 7. In order to enable further manipulation of the flow rate factor to optimize the concentration performance of our device, the suction flow rate at outlet A was controlled by a syringe pump (KDS210, KD Scientific). To show the effect of the suction flow rate on the flow characteristics at the outlet trifurcation, the numerical simulation was conducted (Fig. S3). Figure 6 shows the concentration performance based on different flow rate factors. The suction flow rates at the center outlet were determined based on the simulation results in Fig. S3. As shown in Fig. 6(a), center-focused candida flowed to outlet A at a suction flow rate of 14 μl/min (FF = 7), which was equal to the designed ratio. When the FF was increased to 10 (suction flow rate = 10 μl/min), all the candida was still collected at outlet A. However, when FF was further increased to 17.5 (suction flow rate = 5.7 μl/min), a few of the center-focused candida could not be recovered at outlet A and deflected into the side channels.

Figure 6(b) shows the flow rate factor-dependent concentration factor, which is defined as the ratio of cell concentration of the sample collected at outlet A to the initial cell concentration at the inlet. The concentration of candida depending on different flow rate factors is determined by the suction flow rate from outlet A. An increase in the flow rate factor from 7 (suction flow rate = 14 μl/min) to 10 (suction flow rate = 10 μl/min) increased the concentration factor to approximately 9.9. The concentration factor continued to exceed 9.5 when the flow rate factor increased to 11.7. However, as the flow rate factor increased further (FF = 14.2 and 17.5), the concentration factor decreased to 7.5 and 6.1, because a certain amount of candida flowed to outlet B. Therefore, the optimal flow rate factor was 10 in this study with respect to the concentration of candida. The concentration of the fluorescent-dyed candida was 2.5 × 10^7^ cells/ml in the inlet sample prior to the separation process. After separation, the amount of fluorescent candida was 2.4 × 10^8^ cells/ml in the sample collected from outlet A. The sample collected after the 1st concentration was used in our device for the 2nd concentration process. The final concentration of candida was increased to 2.3 × 10^9^ cells/ml, and this corresponded to a 92-fold increase in the concentration. For PCR analysis, the initial sample volume of 500 μl was reduced to the final volume ~5 μl at the optimal flow rate of 100 μl/min and the flow rate factor of 10, which was processed in ~6 min. In our previous work on malaria parasite separation using the two-stage device^14^, malaria parasites were separated from WBCs with a 94% separation efficiency, 99% purity, and 7-fold concentration increase at 100 μl/min. In comparison, candida cells were separated with 99.1% separation efficiency and 97% purity, and concentrated by 9.9-fold in one-time device processing at the same flow rate (100 μl/min).

### Validation of the device performance

**Figure 7:**
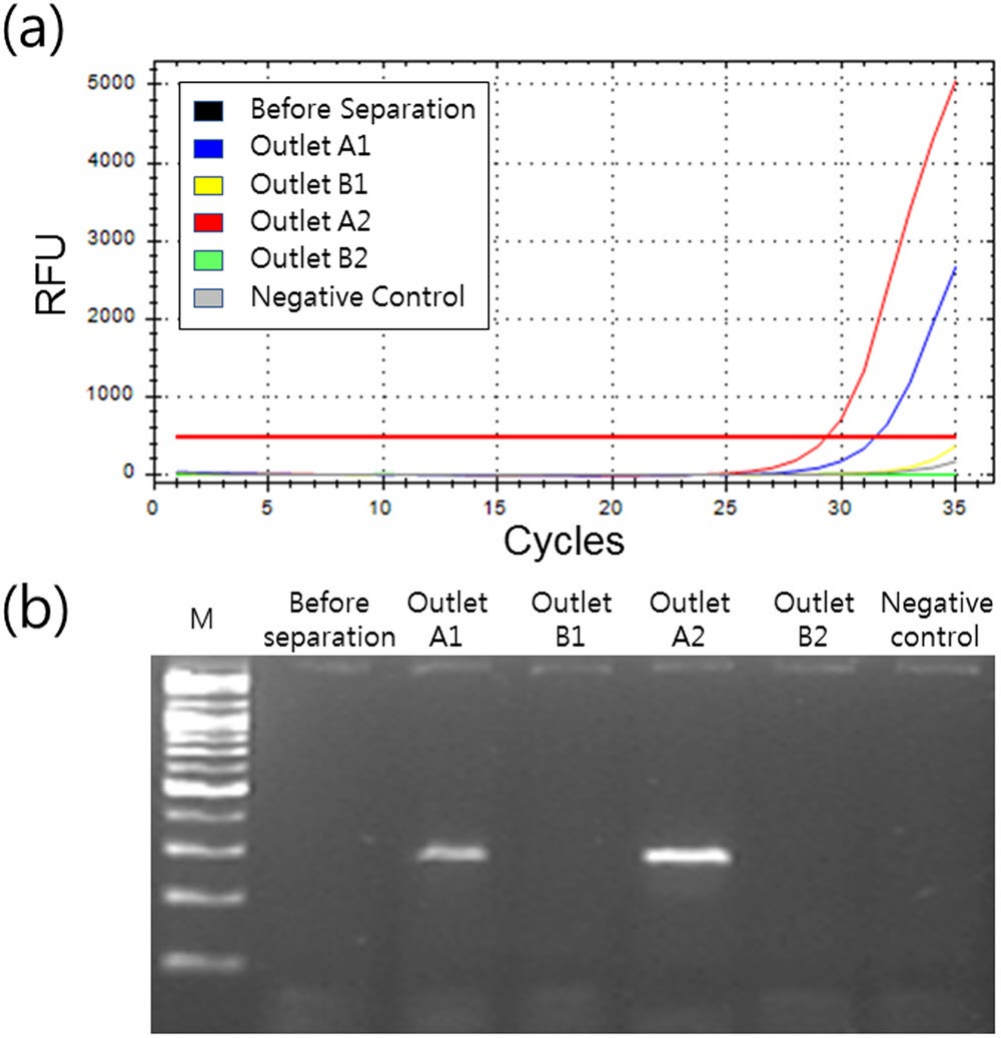
(**a**) Real time amplification curve profile, and (**b**) gel electrophoresis for *C. albicans* samples collected from the device. The numbers marked after outlet A and B indicate the processing number in our device. Full-length gels of (**b**) are presented in Fig. S5.

In order to evaluate the capability of the device to detect *C. albicans*, SYBR green quantitative RT-PCR and conventional PCR analyses were conducted using an undetectable concentration (10^1^ CFU/ml, Table S1) of candida cells in blood. As shown in Fig. 7(a,b), *C. albicans* was barely detected due to the low candida cell numbers and the high number of contaminants, such as WBCs (Ct = 35), prior to the separation. Outlet A1 and outlet B1 indicated the center and side outlets from the 1st process in our device while outlet A2 and outlet B2 indicated outlets in the 2nd concentration process. After the separation and the concentration process, Ct values for the samples collected at outlet A1 and outlet A2 were measured as 31.43 and 29.30, respectively. The samples collected at outlets B1 and B2 and a negative control (Human serum gDNA) were not detected in the PCR condition (Ct = 35). Figure 7(B) shows the results from agarose gel electrophoresis of *C. albicans* samples collected from the device. The amplicon band (approximately 273 bp) of outlet A2 exhibited a stronger band relative to that of outlet A1. This was due to the concentration of candida cells by depleting WBCs and removing the additional buffer solution at outlet B. Furthermore, when the candida cells in the blood were concentrated without leukocyte removal, 10 X blood samples showed lower amplification than 1 X blood samples in PCR, despite the high concentrations of candida cells (Fig. S4).

## Discussion

As shown in Figs 2 and 3, particles with different sizes migrated toward each equilibrium position over a wide range of flow rates from 20 μl/min to 100 μl/min. These results implied the flow rate-insensitive nature of particle patterning in the low-AR channel. Therefore, size-dependent particle migration to the equilibrium positions and particle separation was achieved by non-powered manual operation and negated the external flow injection system with accurate flow rate control.

Compared to our previous works^14,49,50^, simultaneous separation and concentration of candida cells were successfully achieved in the device used in this study. Previously developed devices enabled sensitive size-dependent separation. However, it was difficult to concentrate cells by removing additional buffer solution based on the slight difference between lateral displacements. In addition, in the previous devices, platelets in blood samples could be activated by wall shear stress during initialization of all cells at the 1st bifurcation. However, in the device used in this study, it is assumed that shear-induced platelet activation will not be detected because there is no cell initialization process along the channel walls.

In our device, HA solution was chosen as the viscoelastic fluid with low viscosity (0.9 mPa·s) to achieve the high-throughput processing, compare to PEO solutions used in previous reports (2.3 mPa·s^51,52,54^ and 4 mPa·s^53^). Due to its cost effectiveness, it can be helpful to use PEO solution for the high-throughput processing in our device. However, the effect of molecular structure of PEO and HA solution on the viscoelastic flow has not been examined yet. Therefore, as further study, it is worth examining the flow characteristics using PEO solution by modulating the polymer concentration and the flow conditions.

The capability of high-throughput processing was examined by increasing the flow rate further to 500 μl/min by 100 μl/min interval for candida separation. The throughput of our device can be increased up to 400 μl/min with high separation efficiency (*η* = 98.8 ± 0.6%), which is due to flow-rate insensitivity. This was constrained by the burst of unstable tubing interconnection at the inlet at 500 μl/min^58^. However, according to the previous report, it can be confirmed that the viscoelastic focusing could be achieved at higher flow rate (~20 ml/min) using a rigid chip made of epoxy resin^59^. The microchannel used in previous research was with the width and the height of 80 μm, and the length of 35 mm, which had ~37-fold smaller flow resistance compared to our device. Therefore, our low-AR channel device can be fabricated in rigid thermoplastic to address the present limitation for device mass production during commercialization process. Moreover, the device throughput can be further enhanced by stacking or multiplexing the devices in parallel^60–62^, since our device is comprised of a straight channel with a single inlet without introducing any sheath flows.

As with platelets, there are also cells and residues of various sizes in biological samples such as blood, urine, and the cerebrospinal fluid. Based on the results shown in Figs 2, 3 and 4, particles with various sizes laterally migrated toward the size-dependent equilibrium positions. Particles/cells with the blockage ratio of 0.08 ≤ *β* ≤ 0.2 were tightly focused at the center of the microchannel with the aspect ratio of 0.5, while particles/cells with the blockage ratio of *β* ≥ 0.4 were patterned into two separate streams. Therefore, the capability of our device can be further expanded to manipulate cells and residues of different sizes, for example, separation and simultaneous concentration of malaria parasites (1.5–2 μm), bacteria (0.5–2 μm), circulating tumor cells (15–25 μm), etc., from WBCs.

## Materials and Methods

### Device design and fabrication

The microfluidic channel consisted of an inlet and two outlets. The width and the height of the main channel were 50 μm and 25 μm, respectively, such that the aspect ratio (AR) of the channel was defined as 1/2 (AR = height/width). The length of the main channel was 4 cm. The width of the expansion outlet region was 700 μm to visualize the flow streams of cells.

A polydimethylsiloxane (PDMS) microfluidic channel was fabricated using a soft lithography technique. A replica mold was fabricated using an SU-8 negative photoresist (MicroChem, Newton, MA) on a silicon wafer. The PDMS base and curing agent were mixed at a ratio of 10:1 (Sylgard 184, Dow Corning, USA), degassed in a vacuum chamber, and thermally cured in an oven for 1 h at 80 °C. Subsequently, the cured PDMS channels were peeled off from the replica mold, cut, and bonded on a glass slide with oxygen plasma (CUTE, Femto Science, South Korea). To minimize non-specific protein binding, surface modification was applied to the PDMS channel walls using Triton X-100 surfactant^63^.

### Sample preparation

As a viscoelastic non-Newtonian fluid, hyaluronic acid (HA) sodium salt (357 kDa, Lifecore Biomedical) in phosphate-buffered saline (PBS) at 0.1 (w/v)% was prepared. The zero-shear viscosity and relaxation time of 0.1% HA solution were reported as 0. 9 mPa s and 0.26 ms, respectively^59^.

Fluorescent polystyrene particles with 1 μm (green, G0100, ThermoFisher), 2 μm (blue, B0200, ThermoFisher), 5 μm (green, G0500, ThermoFisher), 10 μm (green, G1000, ThermoFisher), and 13 μm (red, 36-4B, ThermoFisher) diameters were used to examine flow characteristics and verify device performance prior to their application to the candida samples. The particle diameters were selected by considering the sizes of target cells. The particles were suspended in 0.1% HA solution at a final concentration of approximately 2.1 × 10^7^ particles/ml.

Fresh human whole blood with an anticoagulant, ethylenediaminetetraacetic acid (EDTA), was obtained from healthy volunteers at Korea University Guro Hospital (Seoul, Korea) with informed consent from all subjects. Human whole blood samples were obtained from Korea University Guro Hospital complied with safety regulations through an Institutional Review Board (IRB) approved collection method (2017GR0769, approved by Korea University Guro Hospital). The hematocrit of the whole blood sample was ~48%, which was measured using a Hematospin (Hanil Scientific Inc. Korea). *C. albicans* SC5413 was provided by Dr. Jeong-Yoon Kim at the Department of Microbiology & Molecular Biology, Chungnam University, Korea. Yeast were cultured overnight at 30 °C in 10 ml of yeast extract-peptone-dextrose (YPD) broth (Qbiogene), and the cultured cells were then quantified with phase-contrast microscopy (40x power) by using a counting grid. For the application of candida separation, 100 μl of whole blood was mixed with 700 μl 1x BD FACS lysing solution (BD Biosciences), 100 μl of 1x SYBR Green and 100 μl of 1% HA solution. Therefore, the final concentration of HA solution was 0.1%. To reduce the effect of cell-cell interaction which can degrade the separation efficiency, RBCs in whole blood sample were required to be lysed. The concentration of WBCs was approximately 5 × 10^6^ cells/ml. *C. albicans* was spiked at approximately 2.5 × 10^7^ cells/ml, which is a relatively high concentration compared to those in clinical cases, to examine the possibility of clinical applications.

### Experimental procedure

The sample solution was infused into a microchannel by using a syringe pump (LSP01-1A, Longer Precision Pump). During the experiment, flow of particles and cells in the microchannel were observed by an inverted microscope (CKX41, Olympus) with a high-speed camera (V611, Phantom) and a fluorescent camera (CS230B, Olympus). In order to evaluate the separation performance of the device, the inlet sample and each sample from two outlets were quantitatively analyzed using a flow cytometer (Accuri C6, BD Bioscience, CA).

### SYBR Green quantitative RT-PCR and conventional PCR

DNA was extracted from whole blood as described in previous reports with minor modifications^64^. Erythrocytes were briefly lysed in Red Cell Lysis Buffer (Invitrogen, USA) for 10 min at 37 °C. After centrifugation at 3,000 rpm for 10 min, the pellets were treated with 200 μl of 1 M Sorbitol with 5 U/μl of Zymolyase (Invitrogen) at 37 °C for 30 min. DNA from each sample was extracted using the QIAmp DNA Mini Kit (Qiagen) following manufacturer instructions. Real-time (RT) PCR was performed using a SYBR Green Kit (Bio-Rad). For *C*. *albicans* quantification, primers specific to the ITS1-ITS2 region of *C. albicans* were used: forward: TTTATCAACTTGTCACACCAGA and reverse: ATCCCGCCTTACCACTACCG^65^. The qPCR reactive mixture contained 2 μl of gDNA, 10 μl of iQ SYBR Green Supermix (2x), 1 μl of forward primer (5 pmol/μl), 1 μl of reverse primer (5 pmol/μl), and RNase-free water to a total volume of 20 μl. RT-PCR was performed using the CFX-96 instrument (BioRad). Cycling conditions were as follows: initial denaturation at 94 °C for 3 min followed by 35 cycles of denaturation at 95 °C for 20 s, and annealing and elongation at 60 °C for 40 s. For conventional PCR, the PCR reactive mixture contained 2 μl of gDNA, 10 μl of iQ Multiplex Powermix (2x, Bio-Rad), 1 μl of forward primer (5 pmol/μl), 1 μl of reverse primer (5 pmol/μl), and RNase-free water to a total volume of 20 μl. Thermal cycling parameters were the same as the above RT-PCR condition.

## Conclusion

In summary, we described a novel continuous cell separation and concentration device by using a viscoelastic fluid to detect extremely rare candida cells. Polystyrene particles with different blockage ratios were used to estimate the flow-rate and size-dependent flow characteristics using the viscoelastic fluid in a slit microchannel. Particles with a blockage ratio lower than 0.08 (*β* < 0.08) were distributed in the microchannel due to the low blockage ratio. Particles 2 μm and 5 μm in diameter were tightly focused at the center of the microchannel, while particles larger than 10 μm were patterned into two fluorescent streams. Therefore, 2 μm and 13 μm particles were successfully separated at 100 μl/min with high efficiency (approximately 97.8%). The optimized condition was used to separate candida cells with a 99.1% separation efficiency and 97% purity at a flow rate of 100 μl/min. The suction flow rate at outlet A was controlled to achieve the maximum concentration factor of 9.9 with a flow rate factor of 10. Finally, undetectable candida cells at an extremely low concentration (10^1^ CFU/ml) became detectable using the RT-PCR analysis by separation and sequential concentration process. Therefore, the device enables continuous separation and concentration processes for the pre-treatment of extremely rare disease-related cells to improve detection sensitivity.

## Supplementary information

Supporting information

## Data Availability

All data generated or analyzed during this study are included in this published article (and its Supplementary Information files).
