# Supplementary material for: Viscoelastic Separation and Concentration of Fungi from Blood for Highly Sensitive Molecular Diagnostics
Source: Sci Rep. 2019 Feb 28;9:3067. doi: 10.1038/s41598-019-39175-5 (PMC6395622; doi:10.1038/s41598-019-39175-5)
Supplement: Supplementary file 1 — Supporting information [file 41598_2019_39175_MOESM1_ESM.docx]

**Supporting information**

**Viscoelastic Separation and Concentration of Fungi from Blood for Highly Sensitive Molecular Diagnostics**

Jeonghun Nam^1,2,^*^,†^, Woong Sik Jang^1,2, †^, Da Hye Hong^1^, and Chae Seung Lim^1,^*

^1^ Department of Laboratory Medicine, College of Medicine, Korea University Guro Hospital, Korea University, Seoul, Korea

^2^ Department of Emergency Medicine, College of Medicine, Korea University Guro Hospital, Korea University, Seoul, Korea

^†^ These authors contributed equally to this work.

* Corresponding author:

**Jeonghun Nam, PhD**

[jhnam77@gmail.com](mailto:jhnam77@gmail.com);

**Chae Seung Lim, MD PhD**

[malarim@korea.ac.kr](mailto:malarim@korea.ac.kr);

**Flow rate-dependent flow characteristics of 1 μm particles**

Flow rate-dependent distribution of 1 μm particles (*β*=0.04) was monitored in the expansion region with varying flow rates between 20 and 100 μl/min. Figure S1 shows that 1 μm particles were pushed away from the side walls and migrated toward the center region of the microchannel in the entire range of flow rates (20–100 μl/min). However, unlike 2 μm particles (*β*=0.08), 1 μm particles were not tightly focused at the centerline of the microchannel due to the small blockage ratio.

**
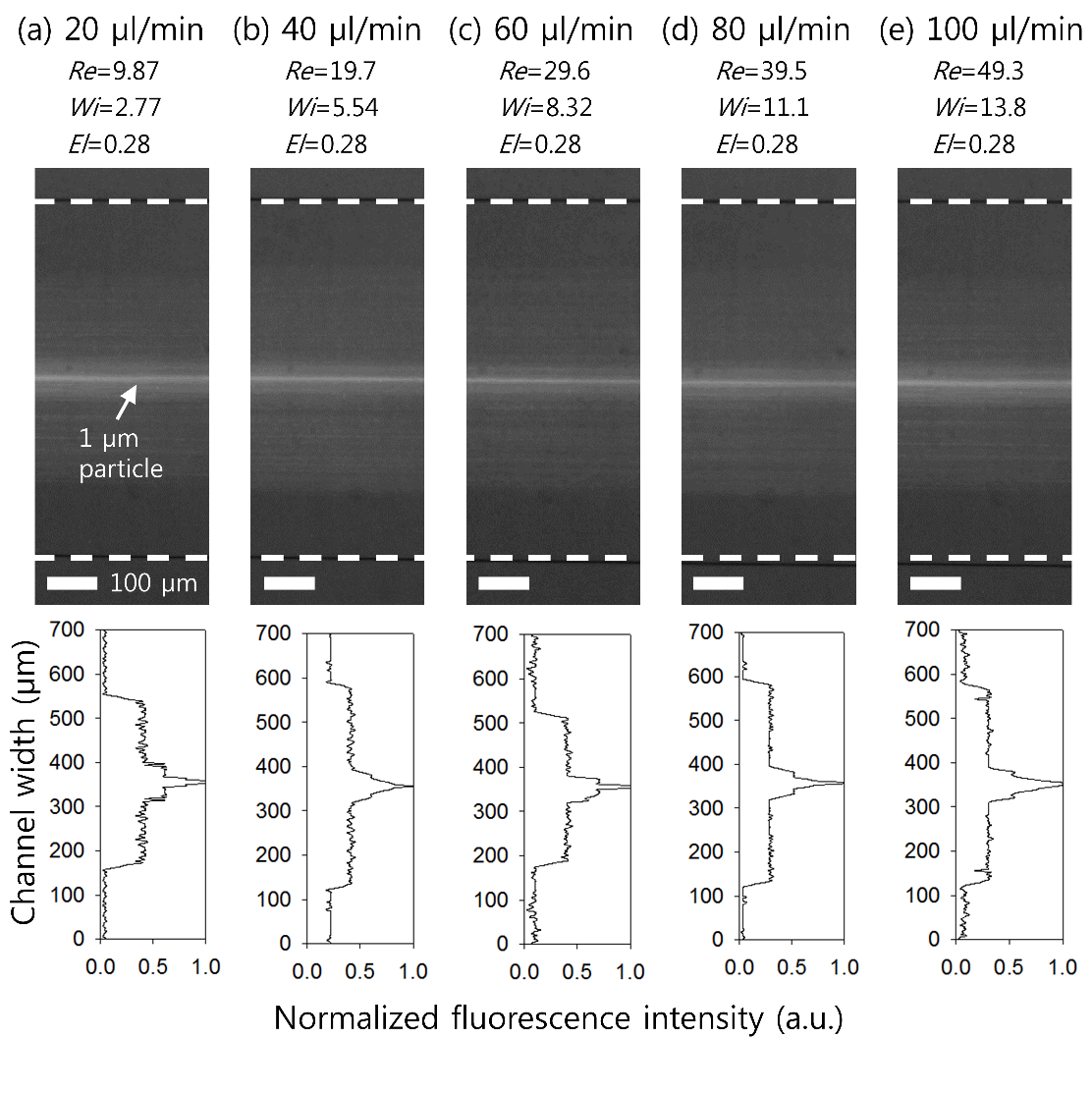
**

**Figure S1.** Flow rate-dependent distribution of fluorescent polystyrene particles with a diameter of 1 μm. Stacked microscopic image (top) and normalized fluorescence intensity (bottom) at different flow rates of 20, 40, 60, 80, and 100 μl/min. The dotted white lines indicate the channel sidewalls.

**Effect of sample solution viscosity on device performance**

The viscosity of lysed blood samples with a hematocrit of 50 is known to be ~ 2.4 cP, which can affect the viscoelastic lateral migration of particles [1]. Therefore, to examine the effect of the viscosity on device performance, aqueous glycerol solution was prepared with viscosities ranging from 1 cP to ~3 cP at ~ 0.25 cP intervals to simulate the viscosity of a lysed blood sample. The viscosities of prepared aqueous glycerol solution were measured using a rheometer (AR2000, TA instruments). Particles with diameters of 2 and 13 μm (*β*=0.08 and 0.52, respectively) were suspended to evaluate the flow characteristics and the device performance on separation and concentration. The lateral position of the particles in the expansion region was quantified as described in a previous study [2]. The expansion region (700 μm width) was divided into 35 virtual segments, each 20 μm wide, and the counted number of particles in each segment was normalized by the total number of particles in the entire region. Figure S2 (a) shows the distribution of particles suspended in 1.5 cP aqueous glycerol solution in the expansion region. Particles 13 μm in diameter migrated toward two off-centered streamlines, while 2 μm particles were focused near the centerline at 100 μl/min, which is almost similar to those in Figs. 2 and 4. However, in glycerol solution with a higher viscosity of 2.75 cP (viscosity ≥ 2.75 cP), two streamlines of 13 μm particles became narrower by approximately 7% to the center, as shown in Fig. S2 (b). In addition, tight focusing of 2 μm particles loosened the particles and some of 2 μm particles migrated out of the separation boundary (dashed red line in Fig. S2). Therefore, the separation and concentration performance decreased slightly. In the center outlet (outlet A), approximately 81% of the 2 μm particles were collected, while approximately 97% of the 13 μm particles were collected from the side outlet (outlet B). Finally, in aqueous glycerol solution with a viscosity lower than 2.5 cP (≤ 2.5 cP), separation of 2 and 13 μm particles could be successfully achieved, so that our device could be applied to separate and concentrate candida cells from a lysed blood sample.


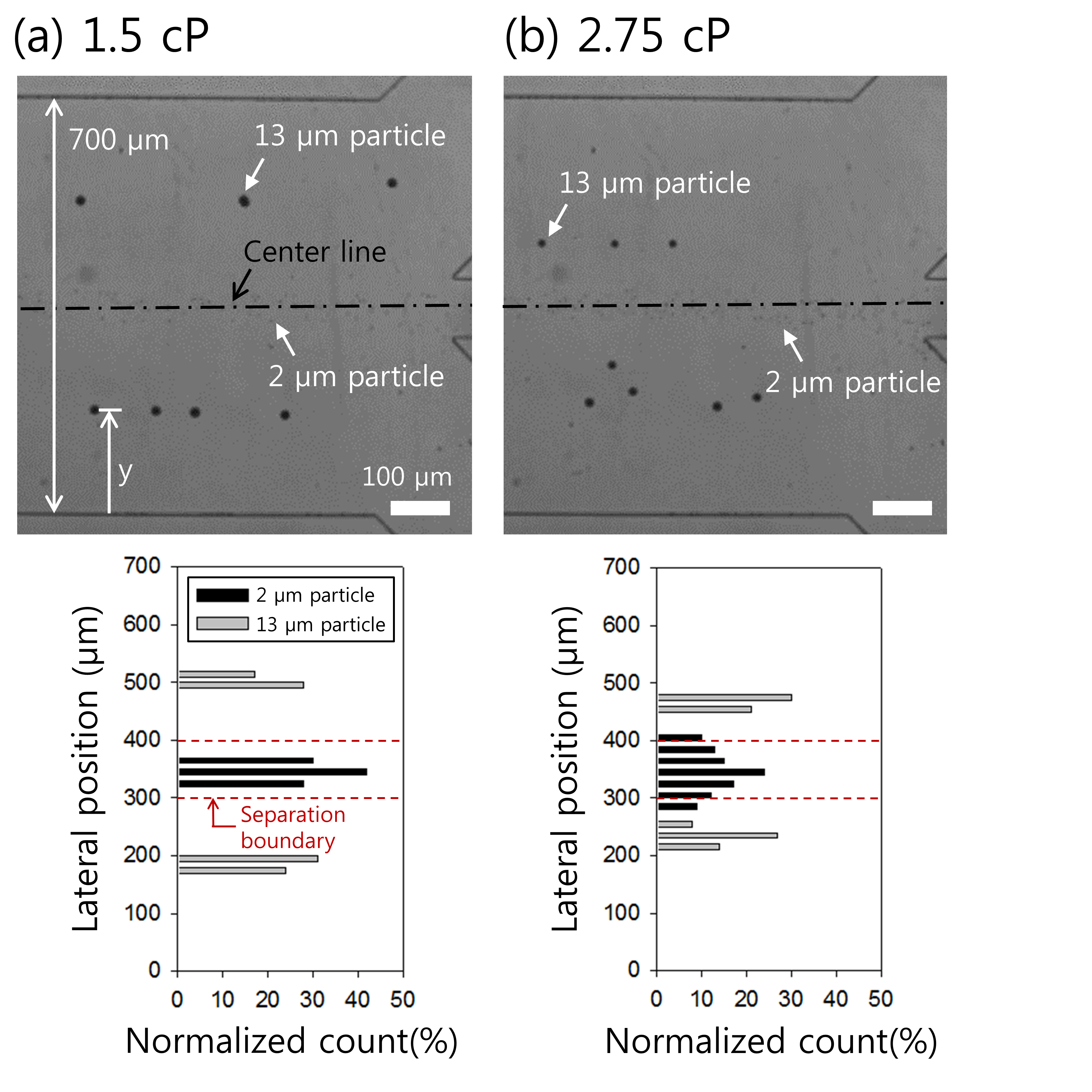


**Figure S2.** Flow characteristics of the binary mixture of 2 and 13 μm particles suspended in (a) 1.5 cP and (b) 2.75 cP aqueous glycerol solution at a flow rate of 100 μl/min. (Top) Stacked microscopic images showing the separation of 2 and 13 μm particles in the expansion region. (Bottom) Lateral position of 2 and 13 μm particles from 35 bins (each 20 μm wide). The dashed red line at the lateral position of 300 and 400 μm indicates the separation boundary.

**Effect of the suction flow rate on the flow characteristics at the outlet trifurcation**

In our device, cells are focused along the centerline of the microchannel in the focused width of w_f_. As the focusing width is tighter, higher separation efficiency can be achieved at the center outlet, outlet A. In addition, by modulating the FF (flow rate factor), additional buffer solution can be removed from the outlet A and the concentration factor can be enhanced. To examine the effect of the suction flow rate on the flow field at the outlet trifurcation, additional experiments and a numerical simulation were conducted.

For flow visualization, we conducted a numerical simulation using the Oldroyd-B model in a straight microchannel. For the simulation, the momentum equation is expressed as:

$$Re\left( u\cdot\nabla\right)u=\nabla\cdot(-pI+\left( {\eta_{s}}/\eta\right)\left[ \nabla u+\left( \nabla u \right)^{T} \right]+T$$

And the extra stress contribution due to viscoelasticity is defined as:

$$T+Wi\grave{T}=\left( {\eta_{p}}/\eta\right)[\nabla u+\left( \nabla u \right)^{T}]_{\nabla}$$

where $\grave{T}=\frac{\partial T}{\partial t}+\left( u\cdot\nabla\right)T-\left[ \left( \nabla u \right)T+T\left( \nabla u \right)^{T} \right]$. These governing equations are non-dimensionalized by using *Re_c_* and *Wi*. Here, *λ* is the characteristic relaxation time, *η_s_* is the relative solvent viscosity, *η_p_* is the relative polymer viscosity, and the total viscosity *η*= *η_s_*_+_ *η_p._*

Figure S3 shows the flow rate factor-dependent flow patterns in the numerical simulation. The inlet flow rate was fixed at 100 μl/min and the FFs were controlled as 5, 7, 10 and 17.5, which had the suction flow rates at the outlet A as 20, 14, 10 and 5.7 μl/min, respectively. With higher FF which means that the suction flow rate at the center outlet becomes smaller, more streamlines went out to the side outlets. Based on the simulation results, the flow rates for suction at the center outlet in Fig. 6 were determined.


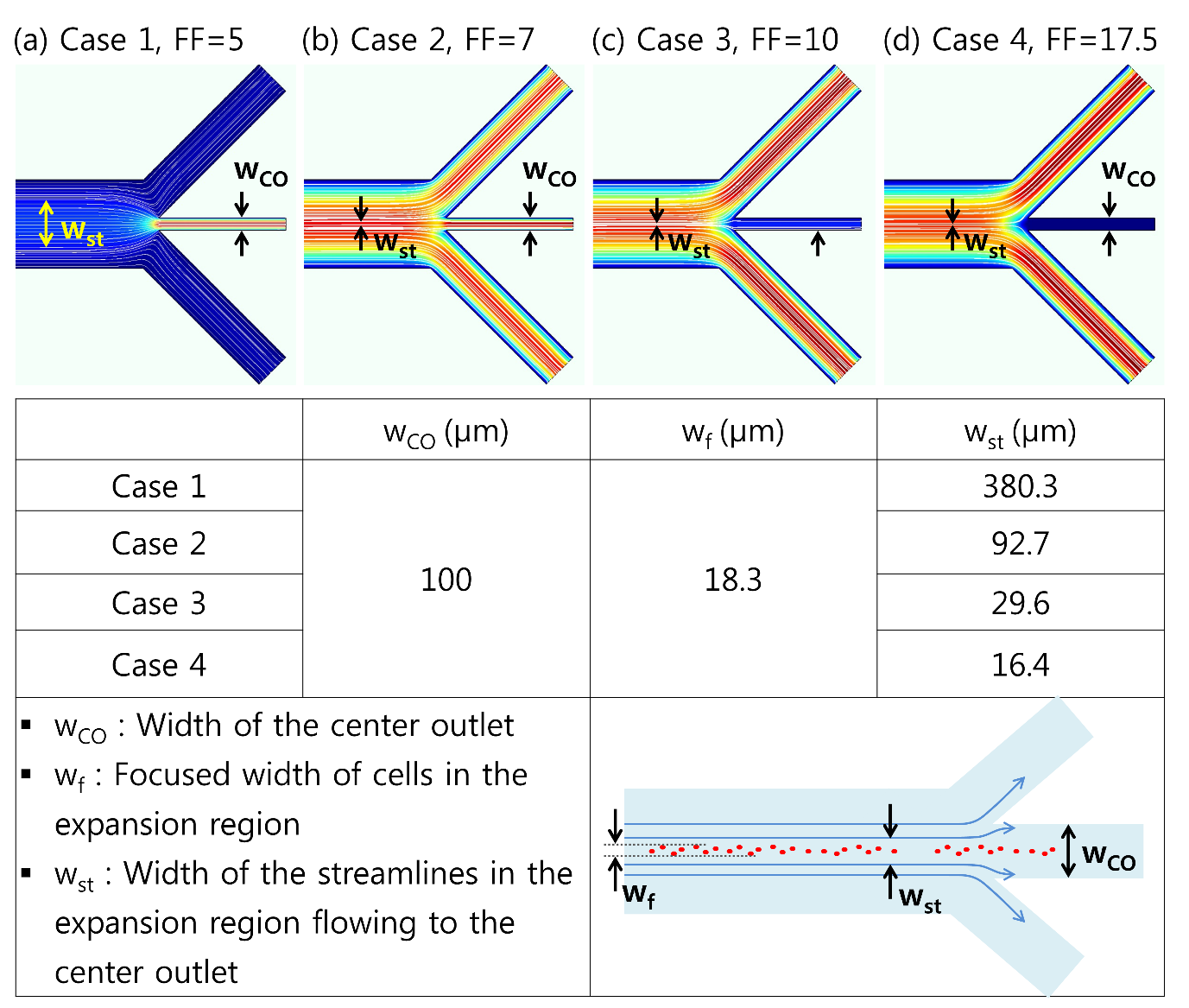


**Figure S3.** Numerical simulation on the flow streamline depending on the flow rate factors of (a) 5, (b) 7, (c) 10 and (d) 17.5.

(a) Case 1: *w_st_* > *w_co_*, *w_st_* > *w_f_*, *w_co_* > *w_f_*

Due to the additional suspending medium, the concentration factor is lower.

(b) Case 2: *w_st_* ~ *w_co_*, *w_st_* > *w_f_*, *w_co_* > *w_f_*

The streamline distribution was same as the width ratio of the outlet trifurcation channels and the concentration factor was similar to the outlet channel ratio.

(c) Case 3: *w_st_* < *w_co_*, *w_st_* < *w_f_*, *w_co_* > *w_f_*

The concentration factor can be increased further by removing additional buffer solution to side outlets.

(d) Case 4: *w_st_* < *w_co_*, *w_st_* > *w_f_*, *w_co_* < *w_f_*

The concentration factor is declined, since the focused cells flow through the side outlets (*w_co_* < *w_f_*).

**Inhibitory effect of white blood cells on the detection of Candida cells in real-time PCR**

To confirm efficiency of candida DNA extraction from candida cells in blood, we compared the limit of detections (LOD) of two DNA samples extracted from PBS and blood spiked with candida cells by using real-time PCR (Table S1). The LOD for PBS spiked with candida cells was 10 CFU/ml, whereas that of blood spiked with candida cells was 100 CFU/ml. This result is consistent with the previous study which showed that the LOD of candida cells in PBS was 10 times higher than that of a blood sample [3]. The difference in LOD between PBS and blood samples spiked with candida cells may be caused by the inhibition of white blood cells (WBCs), since DNA was extracted after red blood cell (RBC) lysis and centrifugation.

Figure S4 shows the PCR analysis results to confirm the inhibitory effect of WBCs. 10 μl of candida cells (10^5^ cells/μl) was spiked in 100 μl of PBS and whole blood, which were *C.* *albicans* in PBS and *C.* *albicans* in 1X blood, respectively. In addition, 10 μl of candida cells (10^6^ cells/μl) was spiked in 1 ml of whole blood, which was *C.* *albicans* in 10X blood. Therefore, the final numbers of WBCs and candida cells in 10X blood were 10-fold of those in 1X blood. After adding 900 μl and 9 ml of RBC lysis buffer to each blood sample (1X and 10X), candida cells and WBCs were centrifuged and applied for the DNA extraction process.

Ct values for the PBS and 1 X blood samples were measured to be 19.53 and 20.41, respectively. However, 10 X blood samples showed higher Ct values (Ct = 33.19), despite having 10 times the concentration of WBCs and candida cells. It was reported that the high quantity of human DNA in whole blood could interfere with primers and probes binding during PCR [4]. The inhibitory effect of whole blood on PCR can be reduced by removing WBCs before DNA extraction or by using methods for specific removal or degradation of human DNA after extraction of nucleic acids [4].

Table S1. Comparison of the limit of detection (LOD) of the real-time PCR using two DNA samples extracted from PBS and blood spiked with candida cells.

| CFU/ml | Ct values | |
| --- | --- | --- |
|  | *C. albicans* in PBS | *C. albicans* in Blood |
| 10^6^ | 19.53 | 20.41 |
| 10^5^ | 23.03 | 24.35 |
| 10^4^ | 25.06 | 27.35 |
| 10^3^ | 29.19 | 30.06 |
| 10^2^ | 31.41 | 33.25 |
| 10^1^ | 33.08 | N/A |
| 10^0^ | N/A | N/A |


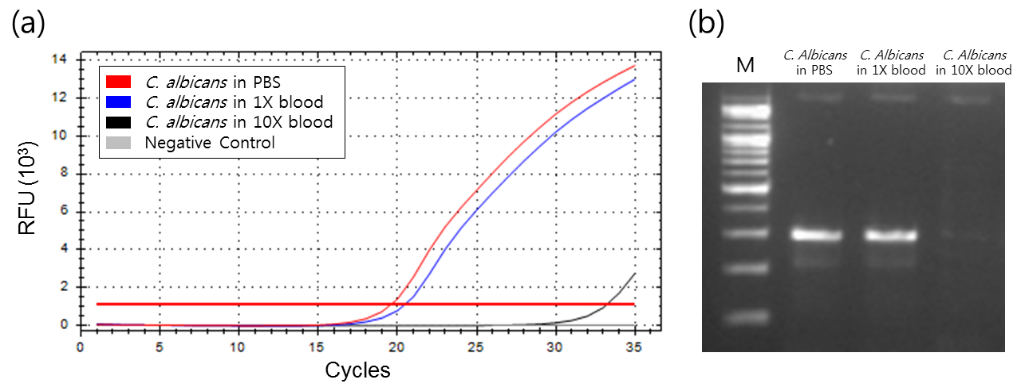


**Figure S4.** (a) Real-time amplification curve profile and (b) agarose gel electrophoresis for DNA samples of *C. albicans* in PBS, 1X blood and 10X blood, respectively. Full-length gels of (b) are presented in Figure S4.


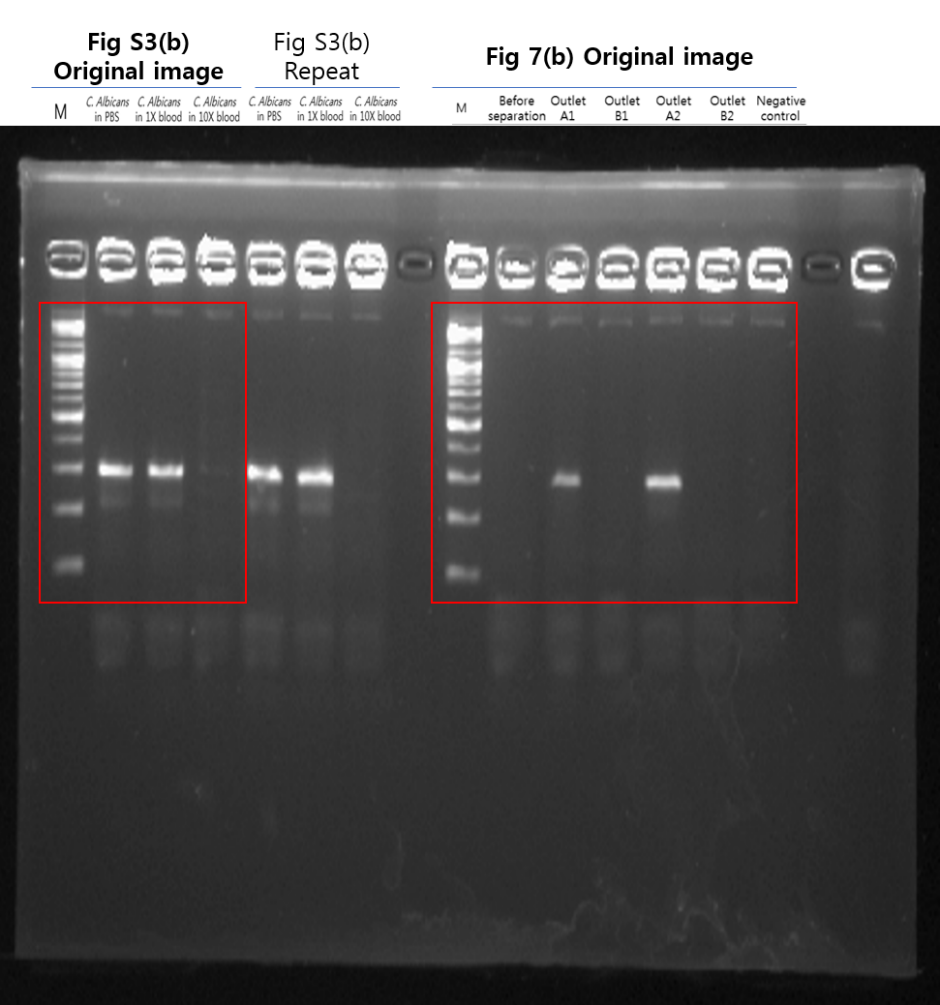


**Figure S5.** The original full-length gel image of Figure 7(b) and Figure S4(b).

**References**

[1] S.Y. Kim, E. Yeom, K. Kim, Korean Soc. Mech. Eng. 12 (2016) 132.

[2] H.W. Hou, A.A. Bhagat, A.G. Chong, P. Mao, K.S. Tan, J. Han and C.T. Lim, Lab Chip, 10 (2010) 2605.

[3] C. Schabereiter-Gurtner, B. Selitsc, M.L. Rotte, A.M. Hirschl and B. Willinger, J. Clin. Microbiol. 45 (2007) 906.

[4] O. Opota, K. Jaton, and G. Greub, Clin. Microbiol. Infect. 21 (2015) 323.
